# Novel p53-dependent anticancer strategy by targeting iron signaling and BNIP3L-induced mitophagy

**DOI:** 10.18632/oncotarget.6233

**Published:** 2015-10-26

**Authors:** Nastasia Wilfinger, Shane Austin, Barbara Scheiber-Mojdehkar, Walter Berger, Siegfried Reipert, Monika Praschberger, Jakob Paur, Robert Trondl, Bernhard K. Keppler, Christoph C. Zielinski, Karin Nowikovsky

**Affiliations:** ^1^ Department of Internal Medicine I, Medical University Vienna, Vienna, Austria; ^2^ Comprehensive Cancer Center, Medical University Vienna, Vienna, Austria; ^3^ Department of Medical Chemistry, Medical University of Vienna, Vienna, Austria; ^4^ Cell Imaging and Ultrastructure Research, University of Vienna, Vienna, Austria; ^5^ Institute of Inorganic Chemistry, University of Vienna, Vienna, Austria

**Keywords:** cancer, p53, BNIP3L, mitophagy, gallium complex

## Abstract

This study identifies BNIP3L as the key regulator of p53-dependent cell death mechanism in colon cancer cells targeted by the novel gallium based anticancer drug, KP46. KP46 specifically accumulated into mitochondria where it caused p53-dependent morphological and functional damage impairing mitochondrial dynamics and bioenergetics. Furthermore, competing with iron for cellular uptake, KP46 lowered the intracellular labile iron pools and intracellular heme. Accordingly, p53 accumulated in the nucleus where it activated its transcriptional target BNIP3L, a BH3 only domain protein with functions in apoptosis and mitophagy. Upregulated BNIP3L sensitized the mitochondrial permeability transition and strongly induced PARKIN-mediated mitochondrial clearance and cellular vacuolization. Downregulation of BNIP3L entirely rescued cell viability caused by exposure of KP46 for 24 hours, confirming that early induced cell death was regulated by BNIP3L. Altogether, targeting BNIP3L in wild-type p53 colon cancer cells is a novel anticancer strategy activating iron depletion signaling and the mitophagy-related cell death pathway.

## INTRODUCTION

The recently developed gallium-based anticancer drug tris(8-quinolinolato)gallium(III) KP46 displays efficient chemotherapeutic activity *in vitro*, has reached clinical evaluation and was successfully tested in a phase I study [1–3]. Though heading toward clinical phase II trials, KP46 activates a variety of complex mechanisms and pathways, converging on cell death of malignant cells in a unique mode that still remains to be elucidated.

Based on the idea that gallium salts may compete with ferric iron [4], impairment of iron dependent cellular pathways and enzymes causing DNA damage and cell cycle arrest was proposed as an anticancer mechanism of gallium-derived drugs [5]. Fe(III) taken up by transferrin mediated endocytosis and released as Fe(II) from endosomal compartments forms labile iron pools (LIP) within the cytosol and other organelles [6]. While not yet proven if KP46 interferes with intracellular iron homeostasis, KP46 was reported to increase the intracellular [Ca^2+^] and mediate Ca^2+^ signaling in p53 dependent and independent apoptosis [7].

Given that *TP53* is the most commonly mutated gene in human cancer [8] we sought to understand the impact of p53 in the cytotoxic mechanism of KP46. Using an *in vitro* colon cancer cell system with p53 wild-type (HCT116^WT^), we present the chronologic events induced by KP46. We identify for the first time the mitochondrial accumulation site of KP46, analyse how KP46 competes with iron and the consequences thereof in respect to the expression of p53 and p53 targets. Highlighting the functions of p53 associated with cell death, we identified the p53-dependent molecular mechanism involved in PARKIN- and BNIP3L-dependent mitophagy, mitochondrial permeability transition (MPT) and mitochondrial cell death pathways induced by KP46.

## RESULTS

### KP46 induces mitochondrial fragmentation, matrix swelling, and accumulates in mitochondria

**Figure 1:**
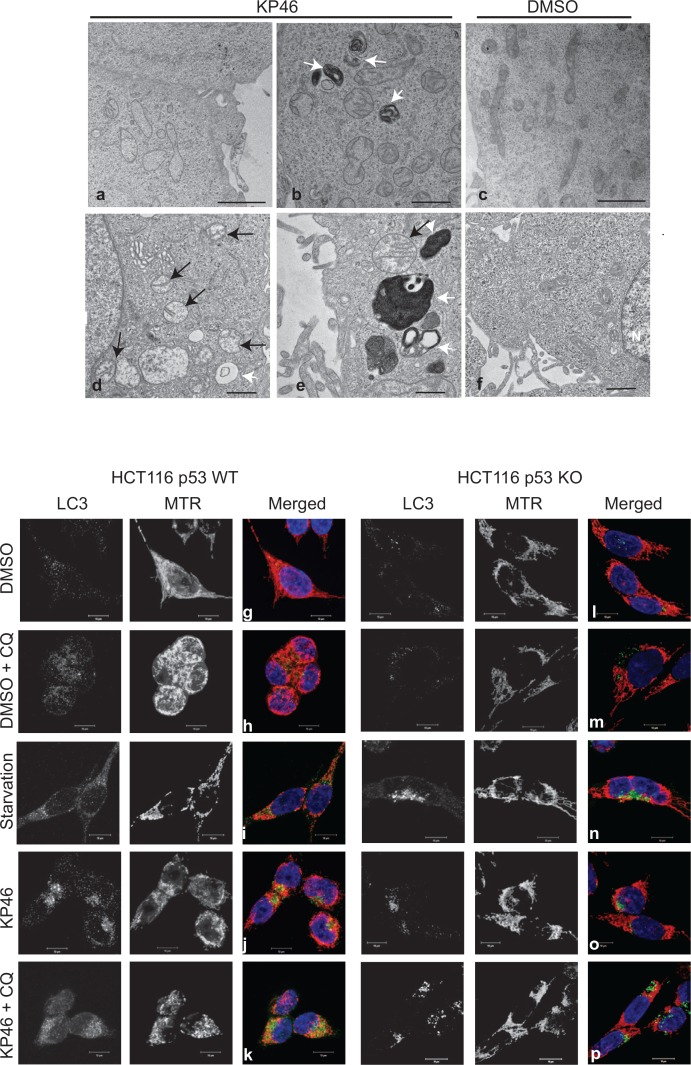
KP46 targets mitochondria a–f Time course electron microscopy was performed on cells treated with 2.5 μM KP46 (a, b, d, e) or with vehicle (c, f) for 4 hours (a, b, c), 24 hours (d, e) and 48 hours (f). White arrows point autophagosomal structures, black arrows mitochondria. Scale bars: 2 μm (a, c), 1 μm (b,d,f) and 0.5 μm (e). **g–p**. HCT116^WT^ (g–k) and HCT116 p53^KO^ (l–p) were exposed to vehicle or 2.5 μM KP46 for 4 hours, in presence or absence of chloroquine (CQ, h, m, k, p) or exposed to starvation for 24 h (I, n), stained with MTR (red) and DAPI (blue), labelled with an antibody against LC3 (green) and visualised by immunofluorescence microscopy. Scale bars: 10 μm.

As revealed by transmission electron microscopy (TEM), HCT116^WT^ cells exposed to KP46 for 4 hours displayed swollen mitochondria with considerably reduced cristae structures (Figure 1a–1b) in comparison to control cells (Figure 1c). The swollen and cristae-poor appearance of mitochondria was persistent and increased in a time dependent manner (Figure 1d–1e) as compared to control cells (Figure 1f). As visualised by confocal microscopy, KP46 disrupted the mitochondrial network and its intracellular distribution (Figure 1j). Interestingly, the perinuclear distribution of the mitochondrial network was dependent on p53, since it was not depicted in HCT116 cells lacking p53 (HCT116p53^KO^) (Figure 1o). We also observed under KP46 conditions the punctuated immunofluorescence of LC3-II, a marker of autophagy (Figure 1j). In contrast to control cells (Figure 1g), the punctuate fluorescence of LC3-II under KP46 increased similarly as under starvation (Figure 1i and 1n) and accumulated in presence of KP46 and chloroquine (Figure 1k and 1p), an agent that blocks endosomal acidification. Interestingly the fluorescence of LC3-II and MitoTracker Red (MTR) colocalised in HCT116^WT^ exposed to KP46 and chloroquine (Figure 1k). Having determined that KP46 targets mitochondria, we assessed the accumulation site of KP46. Taking advantage of the auto-fluorescence of KP46 [9], live imaging of drug treated HCT116^WT^ cells transiently expressing a mitochondrial targeted red fluorescent protein (*mt*RFP) showed the mitochondrial distribution of KP46 (Supplementary Figure S1).

### KP46 induces metabolic insufficiency

**Figure 2:**
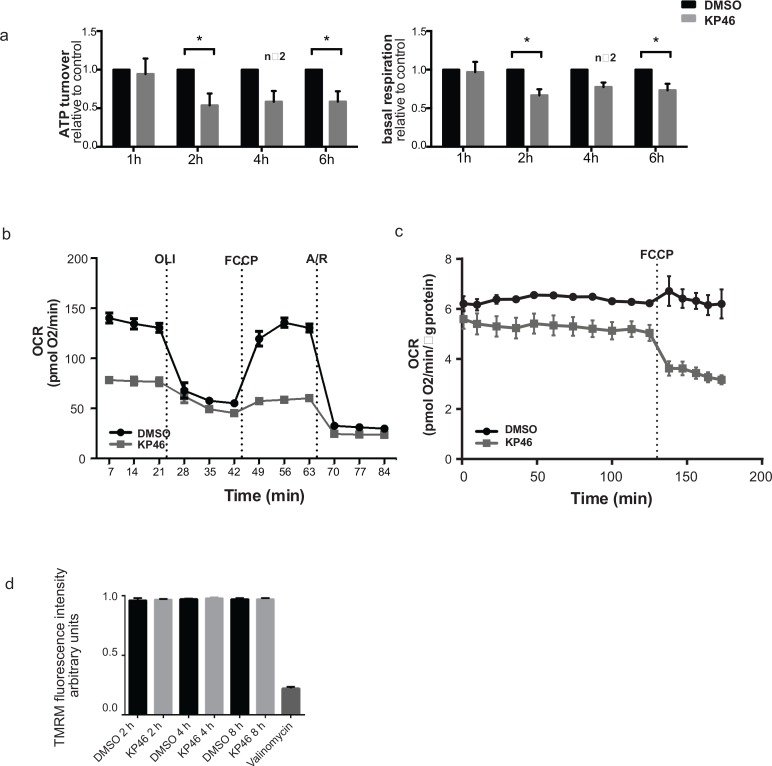
KP46 reduces oxygen consumption by impairing mitochondrial functions **a.** ATP turnover (left) and basal respiration (right) of HCT116^WT^ cells treated with either vehicle or 2.5 μM KP46 for the time indicated. Black bars indicate vehicle while grey bars indicate KP46 treatment, data are means normalized to the vehicle control, error bars represent ± SEM/range (*n* = 3 independent experiments, 4 h-*n* = 2) *p < 0.05, paired *t*-test. **b.** OCR for HCT116^WT^ cells treated with 2.5 μM KP46 or DMSO for 10 hours. Injection ports for sequential additions: OLI: Oligomycin (1 μM), FCCP (0.24 μM) A/R: Antimycin A (0.5 μM) + Rotenone (0.5 μM). Grey lines indicate KP46 treated, black lines vehicle control. OCR was determined as mean value ±SD (*n* = 3). **c.** Oxygen consumption rate (OCR) for HCT116^WT^ cells treated for 6 h with KP46 (10 μM) or vehicle, FCCP (0.2 μM) was added as indicated. Data are means, error bars represent ±SEM (*n* = 4 technical replicates, data are representative of 2 independent measurements). **d.** Flow cytometry analyses of the TMRM fluorescence intensity changes of HCT116^WT^ cells treated with DMSO or 10 μM KP46 for 2, 4 or 8 hours or with 200 nM Valinomycin for 30 minutes. Shown are mean fluorescence intensities. *n* = 3, one-way ANOVA followed by Tukey's multiple comparison test.

To determine if KP46 affects mitochondrial functionality, mitochondrial respiration was measured after short exposure to KP46 or control. Basal respiration and ATP turnover were significantly reduced after 2 hours (Figure 2a). Consistent with these data, the oxygen consumption rates (OCR) were notably decreased upon cell exposure to KP46 after 10 hours (Figure 2b). Moreover, HCT116^WT^ cells treated with KP46 for 4 or 6 hours displayed steadily decreasing OCR over 10 hours post drug exposure (Supplementary Figure S2a–S2b). The simultaneously monitored extracellular acidification rates revealed diminished glycolysis (Supplementary Figure S2c–S2d). To determine if the reduced OCR rates resulted from decreased mitochondrial proteins, nutrient demand or KP46-induced respiratory defects, the carbonyl cyanide-4-(trifluomethoxy) phenylhydrazone (FCCP) induced non-coupled respiration [10] was measured and found to be compromised in KP46 exposed cells as compared to control cells (Figure 2c). Simultaneously, the mitochondrial membrane potential (∆ψ_m_) was monitored. We found that KP46 exposure did not depolarise the mitochondrial membranes (Figure 2d). We concluded that KP46 impaired energy metabolism in a general way and independently of the mitochondrial protein content, ∆ψ_m_ or nutrient demand.

### KP46 downregulates mitochondrial proteins in a p53-dependent manner

**Figure 3:**
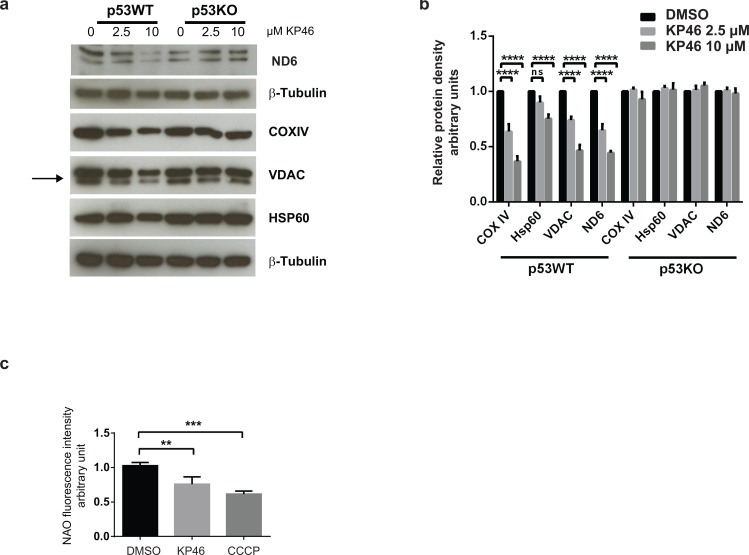
KP46 decreases mitochondrial protein content and mass **a.** HCT116^WT^ and HCT116 p53^KO^ were exposed to vehicle or KP46 2.5 or 10 μM for 6 hours. Protein lysates were immunoblotted with the indicated antibodies. β–Tubulin served as loading control. **b**. Relative protein density of Hsp60, VDAC, COXIV, ND6 normalized to β-Tubulin. *n* = 3, two-way ANOVA, Bonferroni's multiple comparisons test. ****p < 0.0001 **c.** HCT116^WT^ cells were exposed to vehicle, KP46 2.5 μM for 6 hours or 50 μM CCCP for 2 hours, stained with NAO and subjected to flow cytometry. Shown are the mean fluorescence intensities ±SD (*n* = 3), ***p < 0.001, **p < 0.01, one-way ANOVA, Dunnett's multiple comparisons test, ****p < 0.0001.

We next investigated the mitochondrial protein changes caused by KP46 and found decreased expression of the mitochondrial outer membrane protein VDAC, inner membrane proteins ND6 and COXIV while the levels of the matrix heat shock chaperone HSP60 appeared less affected (Figure 3a–3b). The data suggested reduced mitochondrial mass after short term exposure to KP46. In contrast, the mitochondrial protein levels remained abundant and stably expressed in HCT116p53^KO^ under the same KP46 conditions, indicating that the KP46-perturbated mitochondrial protein homeostasis was p53 dependent (Figure 3a).

### KP46 decreases the mitochondrial mass

We asked if KP46-induced early mitochondrial functional/morphological damage and the up-regulation of LC3-II commit impaired mitochondria to removal by autophagy. We quantified the mitochondrial content of HCT116 cells exposed to 2.5 μM KP46 or vehicle for 6 hours, or 50 μM CCCP which induces the autophagic degradation of depolarized mitochondria. Mitochondria labelled with Nonyl acridine orange (NAO) dye (which has previously been used to monitor changes of mitochondrial mass) [11], were monitored by flow cytometry and revealed a KP46-dependent decrease in fluorescence intensity indicative of reduced mitochondrial mass as compared to the controls (Figure 3c). Having shown that KP46 did not decreased the ∆ψ_m_, we can exclude reduced fluorescence intensities of NAO in function of the ∆ψ_m_.

### KP46 drives accumulation of p53 in the nucleus

**Figure 4:**
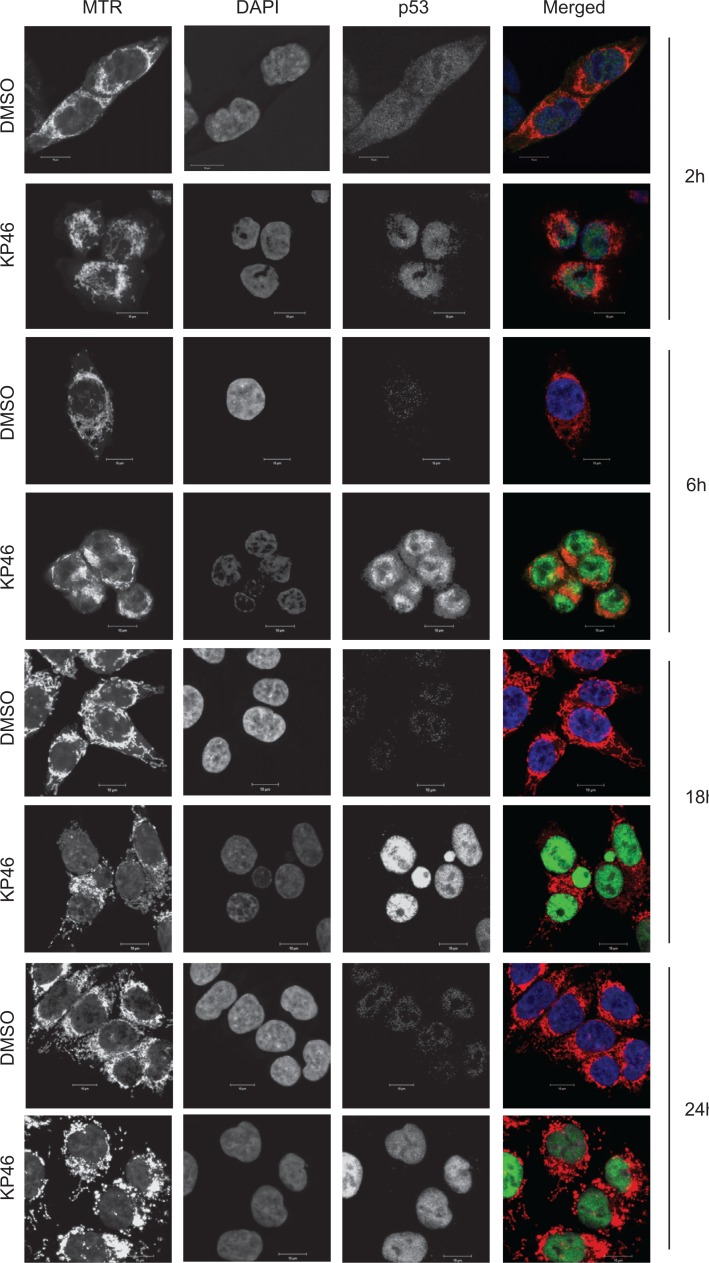
p53 is upregulated in the nucleus Confocal microscopy of HCT116 cells treated with vehicle (DMSO) or 2.5 μM KP46 for the indicated time, stained with MTR (red in the merged Figure), DAPI (blue in the merged Figure) and immunostained with α-p53 antibody (green in the merged Figure). Imaging was performed with identical configuration settings for the 488 channel throughout the time course. Scale bars: 10 μm.

**Figure 5:**
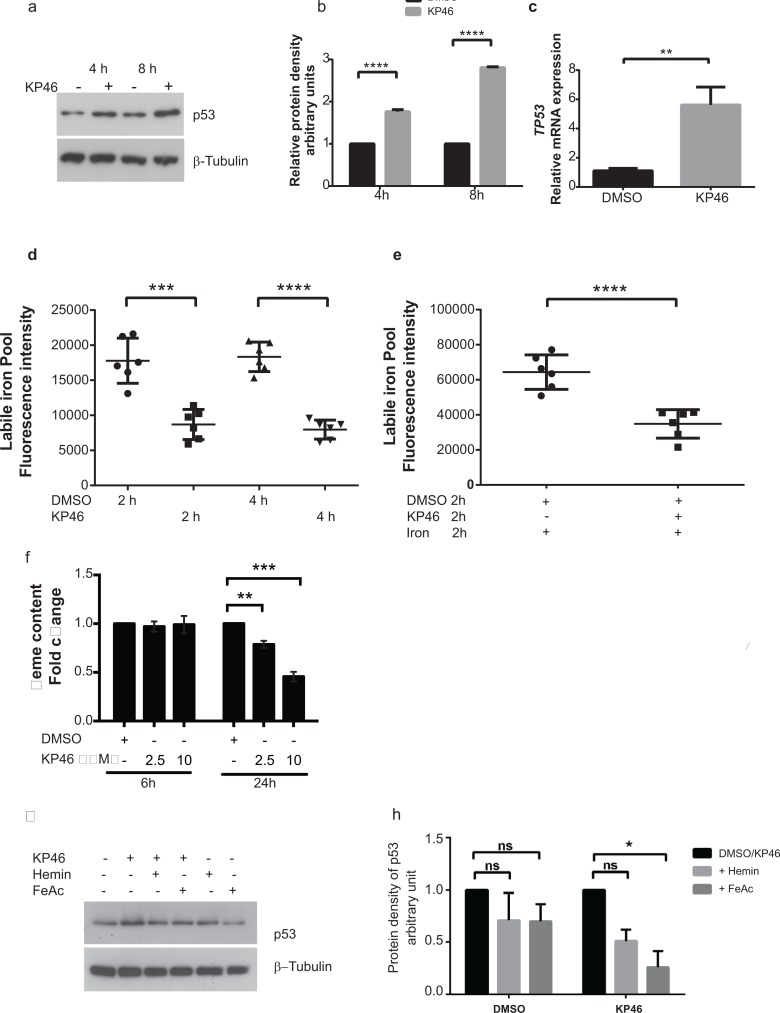
KP46 upregulates p53 in an iron dependent manner **a–b.** Early upregulation of p53. HCT116^WT^ cells were exposed to vehicle or 10 μM KP46 for 4 or 8 hours. **a.** Protein lysates were immunoblotted against anti p53.β-Tubulin served as loading control. **b.** Quantification of the protein density of p53 for 4 and 8 hours relatively to β-Tubulin. *n* = 3, ±SEM, two-way ANOVA followed by Bonferroni's multiple comparisons test, **c.** KP46-changed TP53 transcript expression. mRNA analyses of HCT116^WT^ cells exposed to vehicle or 10 μM KP46 for 4 hours were performed by RT-PCR. Shown is the mean normalized gene expression of TP53, mean values ±SD (*n* = 3 independent measurements, carried out as triplicates), **p-value = 0.0029 using Students *t*-test two tailed unpaired **d–e.** KP46 decreases the labile iron pool. Labile iron pool (LIP) of cells exposed to vehicle or 10 μM KP46 were measured for 2 and 4 hours (**d**) or to iron (FeAc, 600 nM) plus vehicle or plus 10 μM KP46 for 2 hours (**e**) (*n* = 2 independent LIP measurements performed in triplicates). Shown is the mean total fluorescence intensity of calcein measured at Ex485/Em535 nm ±SD. ***p < 0.001 ****p < 0.0001 versus control using one-way ANOVA, two-tailed, unpaired followed by Tukey's multiple comparison test. **f.** KP46 decreases the intracellular heme content. Cells were treated with vehicle or KP46 for the indicated length of time in growth media. Data shown are mean fold changes of protoporphyrin IX relative to the DMSO controls ±SEM (*n* = 3 independent experiments). **p < 0.01,***p < 0.001 using one way ANOVA followed by Dunnett's multiple comparison. **g–h.** KP46 upregulated p53 is reverted by iron and heme. HCT116^WT^ cells were loaded with FeAc (600 μM) or hemin (10 μM) for 6 hours, washed twice with or without DTPA (50 μM), respectively, prior to exposure to vehicle or 10 μM KP46 for 8 hours. **g.** Protein lysates were immunoblotted as in (a). **h.** Quantification of the relative iron- and hemin- mediated protein decrease of p53. *p < 0.05, *n* = 3, ±SEM, two-way ANOVA followed by Bonferroni's multiple comparisons test.

As previously shown for MCF-7 cells [7], KP46 also induced the p53 expression in HCT116^WT^ cells (see Figure 5a–5c). Performing time course microscopy, we observed p53 immunofluorescence gradually increasing after 2 hours and peaking between 12 and 24 hours KP46 exposure (Figure 4). Remarkably, KP46 treated cells displayed an increasing bright staining of p53 exclusively in the nucleus, while the cytosolic staining remained sparse (Figure 4). In contrast, under control conditions, p53 was expressed at sparse basal rate throughout the cytosol and nucleus. Immunoblots confirmed the early KP46-dependent increase of p53 as compared to control cells (Figure 5a–5b). We asked if increased levels of p53 within the nucleus resulted from upregulated transcription, decreased p53 export from the nucleus or both. RT-PCR data confirmed the KP46-induced transcriptional upregulation of p53 (Figure 5c). As recently reported, the small molecule “specific and potent autophagy inhibitor-1” (spautin-1), promotes the turnover of p53 by blocking its deubiquitination via inhibiting the deubiquitinating protease USP10 [12]. However, when co-treated with KP46 and spautin-1, the p53 protein levels remained as abundant (data not shown), suggesting that under KP46, p53 was not exported from the nucleus for ubiquitin-dependent degradation.

### KP46-caused intracellular iron and heme deprivation induces accumulation of nuclear p53

We asked if there was a crosstalk between mitochondrial damage and p53 signal transduction. Recent data revealed that iron depletion strongly upregulates nuclear p53 [13]. Accordingly, we speculated that a decrease of intracellular iron subsequent to KP46 uptake may trigger the upregulation of nuclear p53. To confirm or exclude a signaling role of intracellular [Fe^2+^] in the induction of nuclear p53, we asked if the uptake of KP46 was linked to a depletion of Fe^2+^ pools and measured the changes in the cellular labile iron pools (LIP) immediately elicited by KP46 in HCT116^WT^ cells. The LIP was significantly reduced after 2 or 4 hours KP46 exposure as compared to control cells (Figure 5d). To test whether KP46 reduced the LIP because of competitive uptake with iron, we measured the LIP upon addition of iron in presence or absence of KP46. Addition of iron resulted in high levels of LIP, which were significantly lowered when iron was added concomitantly with KP46 (Figure 5e), underlining the notion of transferrin mediated cellular uptake of KP46 [5]. Thus, depletion of intracellular Fe^2+^ by KP46 was likely a cause of upregulated nuclear p53. As well known, the rate of heme synthesis depends on the intracellular status of Fe^2+^ [14]. Binding of heme to nuclear p53 controls the export of p53 from the nucleus for subsequent degradation [13]. Since the first and last steps of heme synthesis occur in the mitochondria, we asked if heme may function as a signaling molecule between mitochondria and the nucleus and specifically p53. We measured the intracellular heme content of cells exposed to control or KP46 for 6 or 24 hours. While the 6 hours drug exposure did not alter the heme content, significantly reduced levels were recorded after 24 hours (Figure 5f) suggesting a progressive decrease between these two time points.

Next, we tested if preloading HCT116 ^WT^ cells with an excess of iron or heme would prevent the induction of p53 by KP46 and found that iron as well as hemin saturation prior to KP46 exposure blunted the p53 upregulation (Figure 5g–5h). Altogether, we concluded that iron depletion was an early event associated with p53 upregulation in the nucleus. Heme depletion appeared as a secondary effect to iron deprivation, stabilising p53 in the nucleus. Both events resulted in increased accumulation of nuclear p53.

### KP46 triggers PARKIN mediated mitophagy

**Figure 6:**
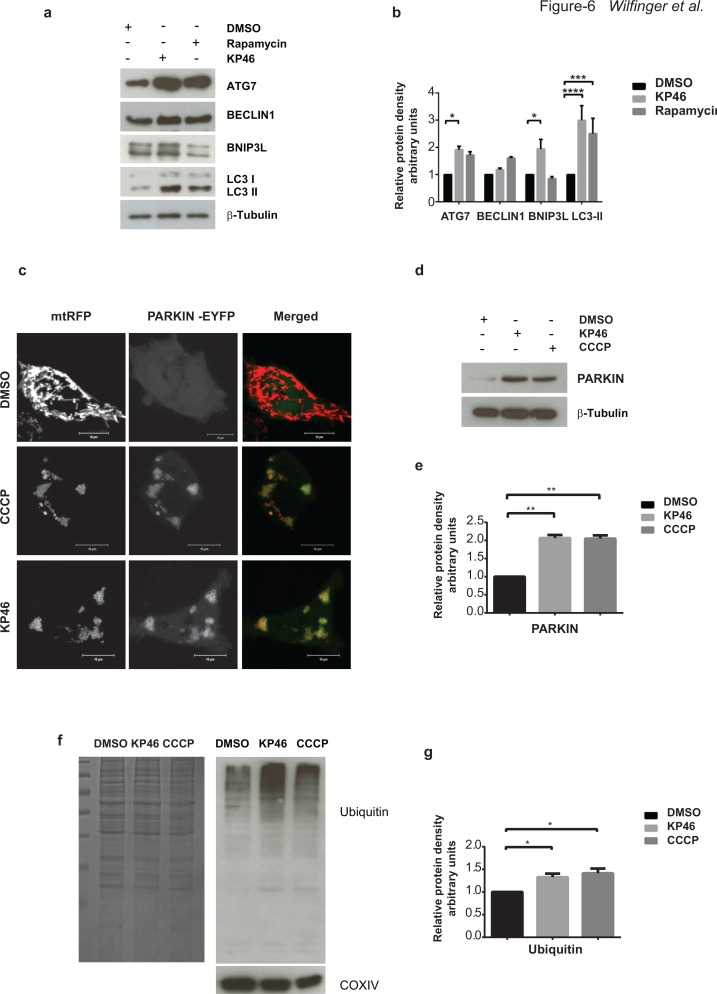
KP46 activates autophagy, PARKIN and ubiquitination **a–b**. KP46 upregulates autophagic markers. a. Immunoblot analysis of LC3, ATG7 and BECLIN 1, BNIP3L. HCT116^WT^ cells exposed 6 hours to vehicle, or 10 μM KP46 or 200 nm Rapamycin. β-Tubulin served as loading control. **b.** Shown are the relative protein densities normalized to β-Tubulin. *n* = 3 (*n* = 5 for Bnip3L in the KP46 treated fraction), ±SEM, two-way ANOVA, Bonferroni's multiple comparisons test. ****p < 0.0001, ***p < 0.001, *p < 0.05 **c.** HCT116^WT^ cells were co-transfected with PARKIN-EYFP (shown in green) and *mt*RFP (red) and exposed to vehicle or 2.5 μM KP46 for 4 hours or 50 μM CCCP as indicated respectively, for 2 hours and immediately monitored under confocal microscopy. Yellow overlay indicates the co-localisation of green and red fluorescence. Scale bars: 10 μm. **d–e.** KP46 activates PARKIN. HCT116^WT^ cells were treated with vehicle, KP46 or CCCP and (**d**) immunoblotted for PARKIN and β-Tubulin and (**e**) quantified. *n* = 3, ±SEM, one-way ANOVA, Bonferroni's multiple comparisons test. **p < 0.01. **f–g.** KP46 triggers ubiquitination of proteins. Mitochondrial proteins were isolated from HCT116^WT^ cells. A gel was stained with Coomassie blue as loading control, and in parallel samples were (**f**) immunoblotted against Ubiquitin and COXIV, as mitochondrial marker, and (**g**) quantified. *n* = 3, ±SEM, one-way ANOVA followed by Bonferroni's multiple comparisons test. *p < 0.05.

As suggested by the findings on mitochondrial defects and mass reduction, we analysed if mitophagy, the selective mitochondrial autophagy was involved in the cell death process. Exploring by western blotting the expression of the general autophagy marker LC3-II, we found low levels of LC3-I and LC3-II in control cells, while KP46-exposed cells displayed as low levels of LC3-I and markedly increased LC3-II, similarly to HCT116 cells treated with the autophagy inducer rapamycin (Figure 6a–6b). Moreover, in contrast to BECLIN1, ATG7 was significantly upregulated under KP46.

However, to specifically study mitophagy rather than general autophagy we sought to study if PARKIN was mobilised to mitochondria. PARKIN is a E3 ligase promoting the ubiquitination of mitochondrial proteins of damaged mitochondria [15] and thus has been involved in “priming” mitochondria for degradation [16]. Previous studies in different cell lines have established carbonyl cyanide m-chlorophenylhydrazone (CCCP) as a strong inducer of PARKIN-mediated mitophagy by uncoupling and depolarizing mitochondria [15, 17]. PARKIN is constitutively expressed in the cytosol under normal conditions and translocates to and targets mitochondria for mitophagy under CCCP conditions [15]. To elucidate if PARKIN recruits compromised mitochondria in HCT116^WT^, cells co-transfected with *PARKIN-EYFP* and *mtRFP* were exposed to CCCP. Live imaging confirmed the recruitment of PARKIN to CCCP-damaged HCT116^WT^ mitochondria. In contrast to a diffuse cytoplasmic distribution under basal conditions, PARKIN-EYFP was detected as highly fluorescent punctae under CCCP exposure (Figure 6c). Similarly to CCCP treatment, we observed PARKIN-EYFP as highly fluorescent punctae associated with and forming rings tightly surrounding mitochondria under KP46 exposure (Figure 6c). Consistently, immunoblots confirmed the upregulation of PARKIN under KP46 to similar level as under CCCP in comparison to control (Figure 6d–6e). General ubiquitination of mitochondria was also confirmed by western blotting (Figure 6f–6g). Furthermore, using *GFP-TAB2 NZF* to visualise K63-polyubiquitin chains [18], we found K63-polyubiquitinated mitochondria (Supplementary Figure S3). The mitochondrial recruitment of PARKIN was an early event as visualised after exposure to KP46 for 2 or 4 hours.

To investigate if mitochondrial degradation was counterbalanced by mitochondrial biogenesis, the transcriptional level of the master regulator of mitochondrial biogenesis [19] PGC1α, was monitored. As compared to control cells, the 4 hour exposure of HCT116^WT^ cells to KP46 significantly downregulated the gene expression of PGC1α. Interestingly, PGC1α remained unchanged in HCT116p53^KO^ (Supplementary Figure S4).

### KP46-induced mitophagy is specifically controlled by p53 and BNIP3L

**Figure 7:**
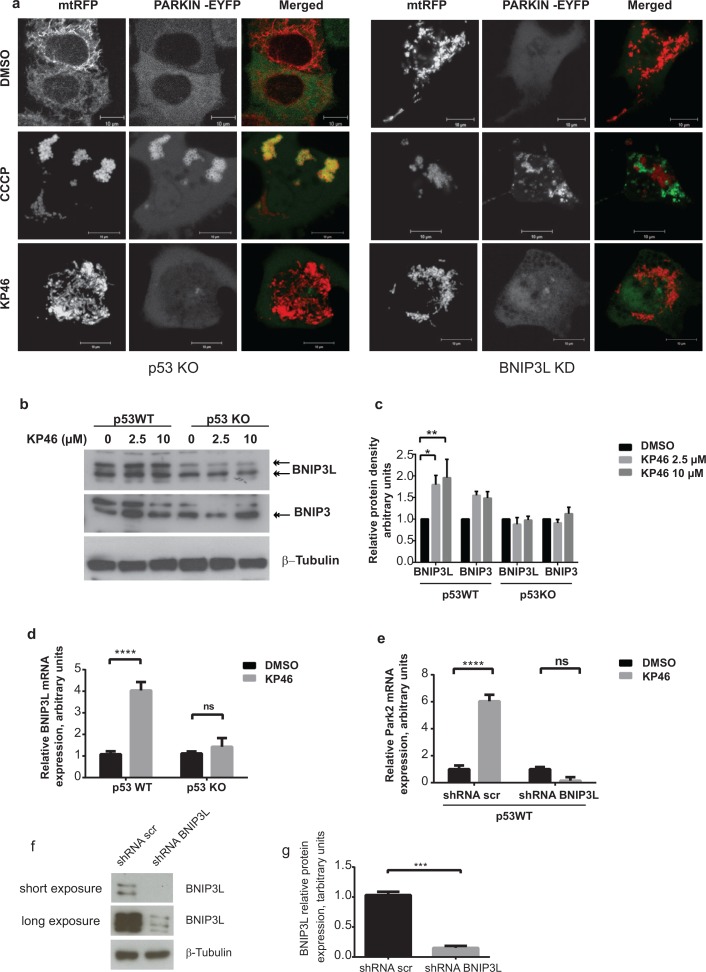
KP46 activates PARKIN in p53 and BNIP3L dependent manner **a.** HCT116p53^KO^ and BNIP3L^KD^ cells were co-transfected with PARKIN-EYFP (shown in green) and *mt*RFP (red) and exposed to vehicle or 2.5 μM KP46 for 4 hours or 50 μM CCCP as indicated respectively, for 2 hours and immediately monitored under confocal microscopy. Yellow overlay indicates the co-localisation of green and red fluorescence. Scale bars: 10 μm. **b-c.** KP46 upregulates BNIP3L in function of p53. **b.** Upregulation of BNIP3 and BNIP3L protein expression. HCT116^WT^ or HCT116p53^KO^ were exposed to vehicle or 2.5 or 10 μM KP46 for 6 hours. Protein lysates were immunoblotted with anti-BNIP3 and anti-BNIP3L antibodies or β-Tubulin (loading control). **c.** Quantification of Bnip3L and Bnip3 expression relative to β-Tubulin. *n* = 3 (*n* = 5 for Bnip3L in p53WT), ±SEM, two-way ANOVA, Bonferroni's multiple comparisons test. **p < 0.01, **p < 0.05 **d.** *BNIP3L* mRNA analyses of HCT116^WT^ or HCT116 p53^KO^ cells exposed to vehicle or 10 μM KP46 for 4 hours performed by RT-PCR. Shown is the mean normalized gene expression of *BNIP3L* ± SD (*n* = 3 individual experiments each in triplicates) two-way ANOVA followed by Bonferroni's multiple comparisons test, ****p < 0.0001 **e.** KP46 induces PARKIN in dependence of BNIP3L. *PARK*2 transcripts were analysed by RT-PCR of HCT116^WT^ cells downregulated for BNIP3L (shRNA BNIP3L) or with scramble shRNA (shRNA scr) and exposed to vehicle (black bars) or 10 μM KP46 (grey bars) for 4 hours. Shown is the mean normalized gene expression of *PARK2* ±SD (*n* = 3 independent measurements, carried out as triplicates). ****p < 0.001, two-way ANOVA, followed by Bonferroni's multiple comparisons test. **f–g.** Downregulation of BNIP3L. *BNIP3L* mRNA analyses of HCT116 expressing shRNA scramble or shRNABNIP3L cells exposed to vehicle or 10 μM KP46 for 4 hours performed by RT-PCR. Shown is the mean normalized gene expression of *BNIP3L* ±SD (*n* = 3 individual experiments each in triplicates), ***p = 0.0005, unpaired Students *t*-test, two-tailed (**f**) and the BNIP3L protein level in HCT116 expressing shRNA scramble or shRNABNIP3L (**g**) and the quantification of the interference, *n* = 3, ±SD, unpaired Students *t*-test, two-tailed, *p* = 0.0002.

To then clarify if PARKIN activation was dependent on p53, we first exposed HCT116 p53^KO^ cells transiently expressing PARKIN-EYFP and *mt*RFP to CCCP. The bright staining of PARKIN-EYFP associated with the fluorescence of *mt*RFP, confirming that CCCP-induced PARKIN was independent of p53 (Figure 7a left panel). Next, we tested if PARKIN was activated by KP46 in absence of p53. In contrast to CCCP-induced mitophagy, PARKIN-EYFP was not recruited to KP46 exposed p53^KO^ mitochondria (Figure 7a). Thus, our data clearly illustrated that p53 was essential for the KP46-mediated PARKIN recruitment to mitochondria. This finding suggested that KP46 triggered p53-caused mitochondrial dysfunctions, which likely induced the mitophagic machinery.

We next asked if activated PARKIN was mediated by nuclear p53 via its transcriptional target, BNIP3L [20]. We found a marked early induction of the BH3-only subfamily proteins BNIP3 and more prominently BNIP3-like (BNIP3L) (Figure 7b–7c). Consistent with the notion of KP46-induced p53 signaling, BNIP3L protein and mRNA levels were not upregulated in isogenic HCT116 p53^KO^ cells treated with KP46 (Figure 7b–7d). Moreover, induction of BNIP3L was also observed at the transcriptional level in HCT116^WT^ while not in HCT116p53^KO^ (Figure 7d).

We further addressed the role of BNIP3L in activating PARKIN in response to KP46. Similar to prior experiments (Figure 6c), we monitored the fluorescence of PARKIN-EYFP upon CCCP in stably downregulated BNIP3L (BNIP3L^KD^) cells (Figure 7a right panel). The addition of CCCP resulted in heavy mitochondrial fragmentation and activation of PARKIN (Figure 7a). However, PARKIN-EYFP was found at a discrete localization from mitochondria. In contrast, KP46 did not activate PARKIN in BNIP3L^KD^ cells, as indicated by low intensity diffused fluorescence of PARKIN-EYFP throughout the cytoplasm (Figure 7a). In line with these results, PARKIN transcripts were robustly induced upon short exposure of HCT116^WT^ cells to KP46 but not in BNIP3L^KD^ cells (Figure 7e) suggesting that KP46 upregulated PARKIN in function of BNIP3L. These data support the idea that BNIP3L was involved in the KP46-caused mitochondrial perturbations, which were responsible for the induction of mitophagy.

### KP46-caused mitophagy is not triggered by mitochondrial depolarisation or reactive oxygen species (ROS)

To study the BNIP3L caused damage to KP46 treated cells we considered decreased ∆ψ_m_, which has been the major factor described in signaling mitophagy. However, as previously shown in Figure 2d (and Supplementary Figure S5a), KP46 did not decrease ∆ψ_m_ within short exposures up to 24 hours, while depolarisation was observed after later time points (Supplementary Figure S5a). Another well documented mitophagy inducer is mitochondrial ROS [21]. Surprisingly, KP46-treated cells did not display any significant superoxide formation at early time points. In contrast, significant superoxide formation was visible after 48 hours and later time points (Supplementary Figure S5b–S5c).

### KP46 sensitizes the Ca^2+^-dependent opening of the mitochondrial permeability transition pore (PTP)

**Figure 8:**
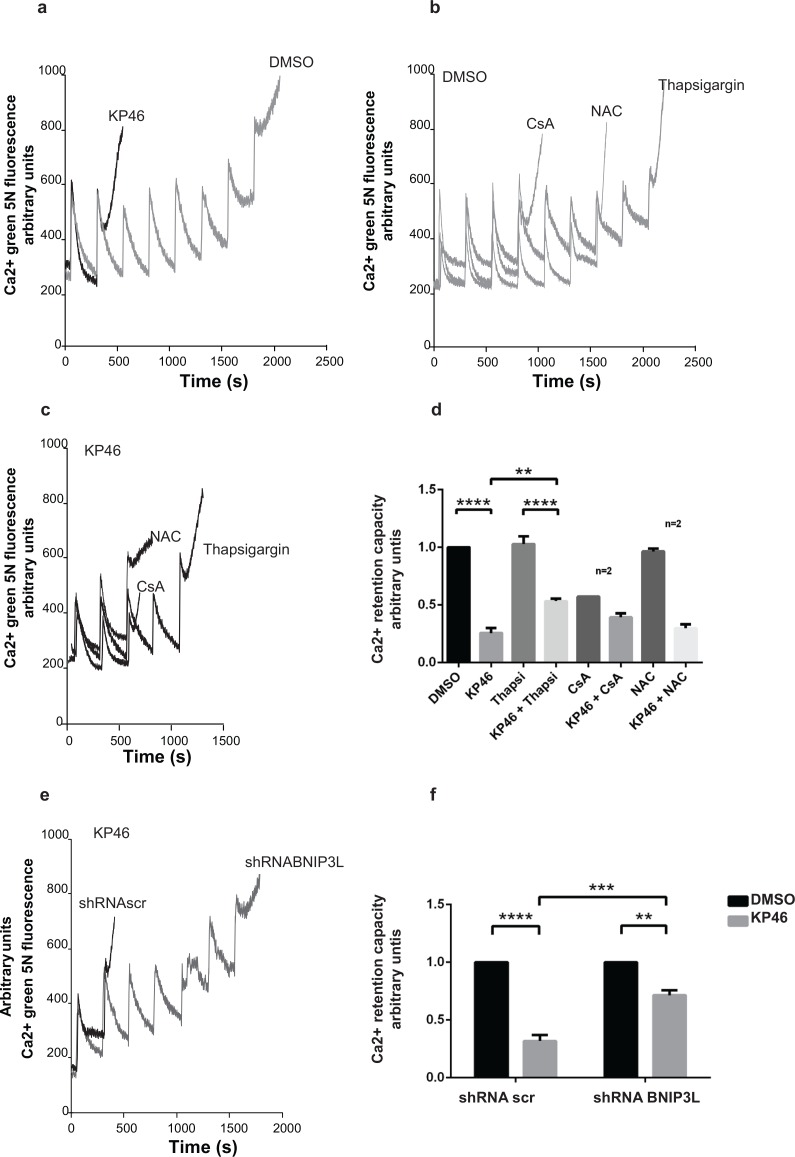
KP46 sensitizes the mitochondrial PTP to Ca^2+^ **a.** CRC was performed on HCT116^WT^ cells exposed to 2.5 μM KP46 (grey trace) or DMSO (black trace) for 4 h. **b–c**. Thapsigargin partly prevents Ca^2+^ induced PTP opening of KP46 treated cells. CRC experiments on HCT116^WT^ cells exposed to 2.5 μM KP46 (b) or vehicle (c), co-treated with CsA (10 μM) added prior measuring or NAC (1 mM) added 1 hour prior and during the KP46 treatment or Thapsigargin (1 μM) added during KP46 treatment. **d.** Quantification of HCT116^WT^ cells treated with 2.5 μM KP46 or DMSO for 4 h with or without the addition of Thapsigargin, CsA or NAC (*n* = 3, if not mentioned separately) ±SEM, ****p< 0.0001, **p < 0.01, two-way ANOVA, followed by Bonferroni's multiple comparisons test. **e.** BNIP3L knockdown prevents PTP opening of KP46 treated cells. CRC were performed as in (a) on HCT116^WT^ transfected with shRNAscr or shRNABNIP3L and treated with 2.5 μM KP46. **f.** Quantification of HCT116^WT^ cells scramble transfected or with Bnip3L KD were treated with KP46 2.5 μM or vehicle for 4 h. *n* = 3, ±SEM, ****p < 0.0001, ***p < 0.001, **p < 0.01, two-way ANOVA, followed by Bonferroni's multiple comparisons test.

We considered increased mitochondrial permeability transition (MPT) as another potential trigger of mitophagy. To investigate if KP46 affected the MPT, we performed a Ca^2+^ retention capacity (CRC) assay, which assesses the maximum ability of mitochondria to accumulate Ca^2+^ until MPT occurs. In contrast to control cells, the threshold level of Ca^2+^ pulses necessary to induce MPT in KP46-treated cells was considerably lowered as indicated by the significantly decreased CRC (Figure 8a, 8e). Interestingly, HCT116 p53^KO^ cells did not display a comparable sensitivity to the PTP opening (Supplementary Figure S6). Confirming that the KP46-sensitized PTP was not caused by ROS formation, co-treatment with the anti-oxidant N-acetyl cysteine (NAC) did not prevent the rapid PTP opening (Figure 8b–8c, 8e). Also cyclosporine A (CsA), a known inhibitor of the pore regulator Cyclophilin-D (CyP-D) had no protective effect (Figure 8b–8c, 8e). However, we found that the addition of thapsigargin, an inhibitor of the SERCA pumps, prevented to some extent the KP46-induced shortening of the CRC (Figure 8b–8c, 8e). These results suggested that KP46 sensitized HCT116^WT^ to the PTP opening in dependence of ER Ca^2+^ stores.

### Ca^2+^-induced PTP sensitivity to KP46 is controlled by BNIP3L

Based on the knowledge that BNIP3L regulates the SERCA pumps [22], we next asked whether KP46 sensitized the PTP in function of BNIP3L and repeated the CRC assays in BNIP3L^KD^ cells. Importantly, BNIP3L^KD^ cells treated with KP46 displayed significantly longer CRC as compared to KP46 treated HCT116^WT^ cells expressing a scramble shRNA (Figure 8d, 8f). These results clearly pointed to the role of BNIP3L in controlling the Ca^2+^-induced MPT of KP46 exposed mitochondria.

### KP46-induced loss of viability is partly reversed by interference of mitophagy

**Figure 9:**
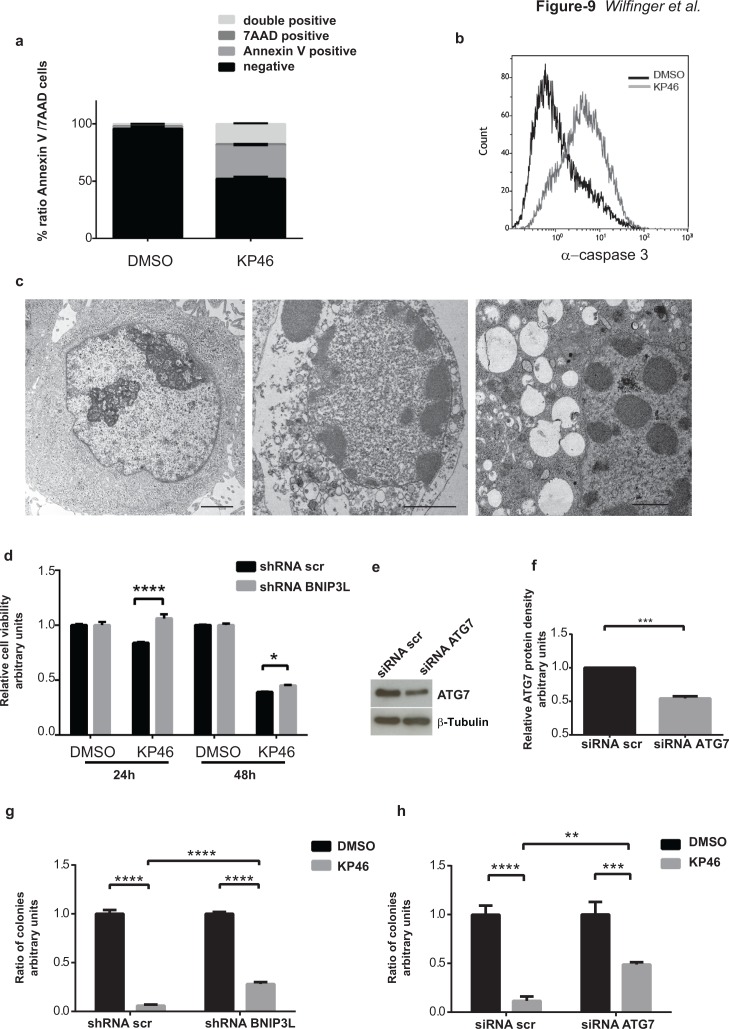
KP46 causes early loss of cell viability and late apoptosis **a.** Early and late apoptotic cells after 48 hours drug exposure. HCT116^WT^ cells were treated with DMSO (left) or 2.5 μM KP46 (right) for 48 hours prior to staining with Annexin V-PE and 7AAD and analyzed using flow cytometry (*n* = 3, ±SEM). **b.** Active caspase 3 in cells exposed to KP46. HCT116^WT^ cells were exposed to 10 μM KP46 or vehicle for 48 hours and the fluorescence intensity of labelled caspase 3 activity was measured. **c.** Ultrastructures of cells exposed for 48 hours to DMSO (left panel) or 2.5 μM KP46 (middle and right panel). Note the structures of secondary necrotic features: condensed nuclear material, segregated nucleoli and the abundance of vesicular structures in the KP46 exposed cells. Scale bars: 2 μm (left and middle panel) 1 μm (right panel). **d.** Viability assay of HCT116^WT^ shRNAscramble and HCT116BNIP3L^KD^ cells. Cells were exposed to vehicle or 10 μM KP46 for 24 or 48 hours. Shown is the relative viability as compared to the respective control experiment (*n* = 3, ±SD) ****p< 0.0001, two-way ANOVA followed by Tukey's multiple comparisons test. **e-f.** Knockdown of ATG7. Cells were transfected with siRNA ATG7 or siRNA scramble and immunoblotted against ATG7 and β-Tubulin (e) and quantified (f) (*n* = 3, ±SEM) Students *t*-test, ***p = 0.0002 **g-h.** Clonogenic assay of HCT116^WT^ shRNAscramble and HCT116BNIP3L^KD^ (g) or HCT116^WT^siRNA scramble and HCT116^WT^ siRNA ATG7 cells (h). Cells were exposed to vehicle or KP46 2.5 μM for 48 hours, sparsely seeded and grown for 14-17 days until colonies formation was detectable. Colonies were counted and quantified. *n* = 3, ±SD, ****p < 0.0001, ***p < 0.001, **p < 0.01, two-way ANOVA followed by Tukey's multiple comparisons test.

In contrast to healthy control cells (HEK293T) exposed to different doses of KP46, the cell viability of HCT116^WT^ was drastically reduced as shown in Supplementary Figure S7a. Although the upregulation of pro-apoptotic proteins was an early KP46-activated p53 response [23], longer exposure to KP46 was necessary to induce late apoptotic features as indicated by the percentage of Annexin V (∼28,52 %) or Annexin V-7AAD double positive cells (∼17,58 %) (Figure 9a). Similarly, caspase-3 cleavage, a general indicator of apoptotic cell death, was significantly detected after 48 hours (Figure 9b). TEM micrographs revealed a highly vacuolized morphology of cells treated with KP46 for 48 hours displaying apoptotic nuclei, condensed nucleoli and features of secondary necrosis (Figure 9c), which were not observed for shorter KP46 exposures. Having demonstrated that interference of BNIP3L blunted mitophagy (Figure 7a) we asked if it would also alter the cell viability. Indeed cell viability was entirely restored after 24 hours as shown by MTT assays (Figure 9d). To investigate longer interference of mitophagy in KP46-induced cell death, cells were treated for 48 hours and clonogenic assays were performed (Supplementary Figure S7b). As shown in Figure 9g–9h, interference of BNIP3L or ATG7 (as shown in Figure 6a–6b significantly increase under KP46) partly protected KP46-exposed HCT116 cells from cell death.

Altogether, the data presented here propose a cytotoxic mechanism of KP46 consisting of targeting an early autophagic cell death type reinforced by a late caspase-dependent cell death pathway, leaving the possibility open that caspase-dependent cell death affects cells escaping mitochondrial clearance.

## DISCUSSION

### KP46 links iron depletion and p53 induction

Concurrently with mitochondrial changes, we detected continuously rising p53 levels as an early response to KP46. p53 did not translocate to mitochondria but consistently remained within the nuclei. Intrigued by the link between the mitochondrial targeting of KP46 and the p53 response, we speculated that there was a correlation between altered iron content and the nuclear p53 expression, as gallium uptake competes with iron, and iron depletion upregulates and iron excess downregulates p53 in the nucleus [13]. Consistent with this idea, we report here that the intracellular uptake of KP46 was accompanied by intracellular iron depletion, which remarkably coincided with p53 activation.

Importantly, p53 binding to heme is required for the export of p53 from the nucleus into the cytosol, where degradation of ubiquitinated p53 takes place [13]. Since mitochondria are major sinks of iron, which they utilize for the biosynthesis of heme and Fe-S clusters [24], heme pools also decrease under iron depletion [13]. Supporting this notion, we found significantly decreased heme content in cells exposed to KP46 as compared to control cells. Corroborating the regulatory role of iron and heme on p53 expression, preloading the cells with iron or hemin prevented the increase of nuclear levels of p53 in KP46-exposed HCT116^WT^. In conclusion, iron and heme depletion are key regulators of the transcriptional expression and nuclear accumulation of p53 under KP46 treatment and furthermore act as the signaling factor between mitochondria and p53.

### p53 status controls KP46-induced mitophagy

This is the first study that identifies mitochondria as the intracellular accumulation sites of KP46. Since mitochondria are important players in determining the survival and cell death commitment of cancer cells, they represent attractive targets of anticancer chemotherapy. As reviewed elsewhere [25], the mostly targeted mechanistic pathways converge on the rupture of the mitochondrial outer membrane, the disintegration of the permeability pore complex and thereby on apoptosis. These mitochondrial cell death pathways are controlled by p53 [26–31] at transcriptional and non-transcriptional levels [26, 27]. Indeed, p53 can also promote apoptosis by translocating from the nucleus to the mitochondrial surface [32] and trigger the permeabilization of the mitochondrial outer membrane by activating pro-apoptotic factors [30, 33] or as recently reported by opening the PTP. p53 also may translocate to the cytoplasm and repress general macroautophagy and mitophagy [26, 29]. However, in the nucleus, p53 regulates the expression of an array of target genes encoding most importantly Bcl-family proteins with pro-apoptotic functions [20, 28, 34].

Here we identified BNIP3L as a major transcriptional target of p53 in response to KP46. BNIP3L belongs to the pro-apoptotic BH3-only Bcl-2 protein family and is a multi-functional protein with critical apoptotic and non-apoptotic (e.g. autophagic) cell death related roles [20, 35]. BNIP3L acts as a mitochondrial stress sensor regulating mitophagy [36], by disturbing the BECLIN1-Bcl2/Bcl-X complex [37]; or triggers caspase-dependent apoptosis through the permeabilization of the mitochondrial outer membrane. Furthermore, it regulates the SR/ER Ca^2+^ pumps, thus increasing the ER Ca^2+^ stores and the delivery of Ca^2+^ from the ER to the juxtaposed mitochondria, eventually inducing programmed necrosis through mitochondrial PTP opening [22, 37]. Consistent with previously described functions, BNIP3L was crucial for PARKIN-mediated mitophagy in the presence of p53. Indeed, PARKIN was not induced or recruited to KP46-exposed mitochondria in cells expressing BNIP3L in the p53^KO^ background or in cells with reduced BNIP3L in the p53^WT^ background, confirming the essential role of BNIP3L in PARKIN signaling. Interestingly, while BNIP3L^KD^ impaired PARKIN-translocation to CCCP-treated mitochondria, BNIP3L^KO^ (Bnip3L^−^/^−^) MEFs entirely failed to mobilize PARKIN under CCCP, underlining its crucial role on priming defective mitochondria for PARKIN [16]. Finally, K63 ubiquitination of KP46-exposed HCT116^WT^ mitochondria underlined the KP46-induced mitophagy-signaling event.

What was the trigger for mitophagy? Our data clearly excluded classical stimuli such as reduced ∆ψ_m_ or ROS [38]. Of note, the serine/threonine-protein kinase PINK1 which is required to activate PARKIN appeared stabilized but not increased under KP46 treated HCT116 as compared to controls (data not shown). Interestingly, iron depletion has recently emerged as a mitophagy signal, however in a HIF1α-dependent and PARKIN-independent context [39]. Another critical signal for mitophagy is mitochondrial swelling as seen under Ca^2+^ overload [40, 41]. Applying external Ca^2+^ pulses induced the opening of the mitochondrial PTP, indicating that KP46 significantly sensitized the MPT. Considering the mitochondria as a delivery site for KP46, we propose that disturbed mitochondrial ion homeostasis induce mitochondrial swelling and thereby mitophagy. Based on data presented here, we may speculate that KP46 induced in a p53/BNIP3L dependent manner the flickering of the PTP that was not strong enough to cause an overall depolarization but enough to signal the initiation of mitophagy and mobilize the mitophagy players, especially PARKIN. Based on previous evidence showing that BNIP3L regulates the ER Ca^2+^ stores [42] and that KP46 increases intracellular Ca^2+^ [7] we propose that upregulated BNIP3L controls the MPT of KP46 treated cells by increasing the transfer of ER Ca^2+^ to the mitochondria. Supporting this idea, we show that thapsigargin, the inhibitor of the ER/SERCA pumps partly prevented the Ca^2+^-induced opening of the PTP while the antioxidant NAC did not. Similarly, BNIP3L downregulation in KP46 treated cells repressed the Ca^2+^-induced PTP opening. Taken together, consistent with a role of BNIP3L in regulating the MPT [22, 43] and mitophagy, we identified BNIP3L as the nuclear target of p53-driven mitophagy [38], ER Ca^2+^ release and eventually cell death upon KP46 exposure.

Remarkably, while PARKIN, BNIP3L and nuclear p53 were upregulated, PGC1α was repressed. In general, mitophagy functions as cell survival strategy by clearing dysfunctional mitochondria. However, survival cannot be sustained under disproportionate mitochondrial clearance and reduced biogenesis. Consequently, mitophagy becomes a cell death process requiring excessive formation of autophagic organelles. Like the pro or anti survival roles of mitophagy, the mechanistic modes of action of mitophagy are intricate as well. Several mechanisms have been described for p53 in negatively regulating mitophagy in function of its intracellular localization under normoxic or hypoxic conditions. Accordingly, while cytosolic p53 has been shown to bind to PARKIN and repress mitophagy [44, 45], translocation of p53 to mitochondria has been reported to protect from mitochondrial dysfunctions and thereby prevent mitophagy [46]. In line with a protective role of mitophagy under stress conditions, very recently it was shown that the accumulation of p53 in tumor cells under hypoxia - by use p53 fused to the oxygen-dependent degradation domain of HIF1α - inhibited PARKIN-mediated mitophagy and thereby increased the radiosensitivity of these tumor cells [47].

In conclusion, demonstrating a crucial role of the p53 status in determining the cell death pathway, we show a novel p53-dependent drug response where p53 accumulates in the nucleus to induce BNIP3L as a main actor and mitophagy as chief factor regulating cell death.

## MATERIALS AND METHODS

### Reagents

High purity KP46 [tris(8-quinolinolato)gallium(III)] was synthesized according to previously described methods [48] at the Institute of Inorganic Chemistry, University of Vienna (Austria). For *in vitro* studies, compounds were dissolved as 4 mM stocks in 0.1% Dimethyl sulfoxide (DMSO) and used at the indicated concentrations. The IC_50_ value for HCT116^WT^ being determined to be 1.06 μM ±0.35 [23], we applied KP46 at 2.5 or 10 μM to study the short term effects on mitochondrial morphology. DMSO (0.1%) is used as vehicle throughout all experiments unless otherwise indicated. Tetramethylrhodamine methyl ester, (TMRM) #T-668, MitoTracker Red CMXRos # M7512, MitoSOX Red # M36008 Calcium green 5N # C-3737, Coomassie brilliant blue G-250 dye #20278 were from Molecular Probes, Life Technologies (Invitrogen, Paisley, UK). Salicylaldehyde isonicotinoyl hydrazine (SIH) was a generous gift from Dr. P. Ponka (Lady Davis Institute for Medical Research, Montreal, Canada). All other substances were from Sigma–Aldrich (St.Louis, USA).

### Cell culture, transfection, plasmids

Human HCT116^WT^ colon carcinoma cells and their isogenic subline with p53^KO^ were generous gifts from Dr. Vogelstein, John Hopkins University (Baltimore, MD). The cells were grown in McCoy's 5A culture medium with glucose (3g/L), 10 %FBS and 1% Pen/Strep at 37°C in humidified atmosphere under 5% CO_2_. TurboFect Transfection Reagent from Fisher Thermo Scientific (#R0531) was used for transfections according to the manufacturer's protocol. The plasmids used for transient transfection were *mt*RFP-pcDNA3, kindly provided by T. Pozzan, University of Padua, Italy, Parkin-EYFP-pZsYellow1-N, by Richard Youle (National Institute of Neurological Disorders and Stroke, National Institutes of Health, Bethesda, MD) and GFP-TAB2 NZF and GFP-TAB2 NZF E685A by Ivan Dikic (Goethe University School of Medicine, Frankfurt/M, Germany). For shRNA-mediated gene knockdown of BNIP3L and scramble controls following plasmids were used: Addgene # 17469 and Origene # TR316466. We established a stable BNIP3L knockdown cell strain with Origene # TR316466 and KD of ATG7 by use of ATG7HSS173705 Invitrogen #5271626.

### Viability assay

The cytotoxicity of KP46 was determined based on a 3-(4,5-dimethylthiazol-2-yl)-2,5-diphenyltetrazolium bromide (MTT) viability assay (EZ4U, Biomedica, Vienna, Austria). HCT116^WT^ cells shRNAscr or shRNABNIP3L transfected cells were exposed to vehicle or KP46 (10 μM). Untransfected HCT116^WT^ cells were exposed to vehicle or KP46 (10 μM) for 24. After treatments, the media were removed and replaced with 100 μL EZ4U-MTT assay solution (1:10 dilution in growth media), incubated 90 minutes at 37°C. The absorbance was measured using a micro plate reader (TriStar LB941, Berthold Technologies) at 450 nm. All experiments were carried out at least twice as triplicates.

### Clonogenic assay

HCT116 p53^WT^, HCT116 p53^WT^Bnip3L^KD^ and HCT116 p53^WT^siATG7 cells were treated for 48 hours with vehicle or KP46 2.5 μM. Thereafter 1×10^3^ cells/well were seeded onto 6-wells plates. Cell specific medium was changed every third day for 14–17 days, washed with 1x PBS, fixed with Methanol/Acetone for 10 minutes, washed as before, stained with Crystal Violet for 10 minutes and washed again. Colonies were counted manually and in parallel by using the ImageJ software. Images were created using the TissueFAXs (TissueGnostics).

### RT-PCR

Total RNA was extracted using TRIzol reagent (Invitrogen) followed by DNA digestion with DNase I, Amplification Grade, Life Technologies (Invitrogen, Paisley, UK) and was then reverse transcribed using the High-Capacity cDNA Reverse Transcription Kit (Applied Biosystems, Foster). Quantitative real time PCR was performed using SYBR reagents (Eurogentech, Seraing, Belgium). The expression levels of genes of interest were normalised to S18. Following primers were used: PGC1α: forward: ACCACAAACGATGACCCTCC, reverse: GTGGAGTTAGGCCTGCAGTT; PARK2: forward: CCATGATAGTGTTTGTCAGGTTCA, reverse: TGGAAGATGCTGGTGTCAGAA; BNIP3L: forward: GGACTCGGCTTGTTGTGTTG, reverse: TCCACCCAGGAACTGTTGAG. Primers for TP53 were described in [49], for S18 in [50].

### Mitochondrial isolation

Mitochondrial isolation was performed according to the protocol of Frezza C. and Scorrano L. [51]. Briefly, cell pellets were resuspended in a buffer (IBc) containing 1 mM Tris-MOPS, 0.1 mM EGTA/Tris, and 200 mM sucrose, pH 7.4, homogenized using a Potter (overhead stirrer, OST basic, #3145000) at 1400 rpm and centrifugated at 600 g for 10 min at 4°C. The supernatant was centrifugated at 7000 g for 10 min at 4°C. The resulting pellet contains the mitochondrial fraction.

### Western blot analysis

Mitochondrial pellets resuspended in IBc or whole cell protein lysates (40 μg/lane) resuspended in lysis buffer: 150 mM NaCl, 50 mM Tris pH7.4, 0.5% deoxycholate, 2 mM EGTA, 5 mM EDTA pH 7.4, except for the enrichment of nuclear proteins where the buffer contained 100 mM Tris-HCl, pH 9.5, 1% SDS, were separated by SDS-PAGE and transferred onto PVDF membranes 0.45 μm (#88518 Thermo Scientific, Rockford, IL, USA). PageRuler Prestained Protein Ladder (# 26616) was from Life Technologies, Thermo Fisher Scientific, (Rockford, IL) was used. Blots were incubated with respective antibodies and the expression levels of proteins determined by use of SuperSignal West Pico Chemiluminescent Substrate (Thermo Scientific, #34080). Antibodies against VDAC/ Porin monoclonal mouse (#14734), β-Tubulin conjugated to HRP (#21058) and BNIP3L (#8399) and Anti-NADH-Dehydrogenase subunit 6 (#81212) were from Abcam, Cambridge, UK, Hsp60 (#1052) Parkin (PRK8, #32282) and p53 (#126) from Santa Cruz Biotechnology Inc, CA, COX IV (#4850), LC-3 (#2631), ATG7 (#3738), BECLIN1 (#2775) Ubiquitin (P4D1, #3936) from Cell Signaling Technology Inc., Beverly, MA., and Bnip3 (B7931# clone Ana40) from Sigma-Aldrich.

### Flow cytometry analysis

To assess cell death, cells exposed to vehicle or KP46 (2.5 or 10 μM, as indicated) for 48 hours were pelleted, washed with 1x phosphate buffered saline (PBS) (137 mM NaCl, 2.7mM KCl, 8 mM Na_2_HPO_4_ and 2 mM KH_2_PO_4_), centrifuged at 600 *g* for 7 minutes at 4°C and transferred into FACS buffer (4% BSA in PBS). Then, cells were stained with Annexin V PE and 7 AAD using the Annexin V:PE Apoptosis Detection Kit I (BD Pharmingen, San Diego, CA, USA #559763) according to the manufacturer's protocol washed and immediately analysed with the Gallios flow cytometer (Beckman-Coulter, Miami, FL, USA). To measure active Caspase 3, cells treated with vehicle or KP46 for 24 and 48 hours were resuspended in the fixing solution for 10 minutes at 4°C, washed and stored in the permeabilisation solution for 15 minutes at 4°C (IntraPrep Fixation and Permeabilization Reagent, Beckman Coulter, 07803). Cells were incubated with the anti-active Caspase 3 rabbit antibody (BD Pharmingen, San Diego, CA, USA #559565) for 30 minutes at 4°C in the dark, washed with PBS, and incubated with the secondary FITC donkey anti-rabbit IgG (Jackson ImmunoResearch, #711-096-152) for 30 minutes at 4°C in the dark, then washed with PBS and analysed by flow cytometry (Beckman Coulter, Cytomics FC500).

To determine the ∆ψ_m_, cells exposed to vehicle or KP46 (10 μM) for 2, 4, or 8 hours, were stained for 30 minutes with TMRM (100 nM). Exposure to Valinomycin (200 nM) for 30 minutes at 37°C served to generate depolarised controls. Cells were washed with PBS, trypsinised and resuspended in fresh growth medium. After two washing steps, the TMRM fluorescence intensity was immediately visualized by Gallios flow cytometer on FL2. Populations with polarised and depolarised mitochondrial membrane potential indicated by TMRM fluorescence intensity were quantified according to their arithmetic mean value.

### Nonyl acridine orange measurements

HCT116 p53^WT^ cells were exposed to vehicle or KP46 for 6 h, trypsinised, centrifuged at 600 g for 7 minutes, washed with 1x PBS, centrifuged as before and fixed with 70% ethanol for 10 minutes. Then, cells were washed once with 1x PBS and stained with 500 nM Nonyl acridine orange (Acridine Orange 10-Nonyl Bromide, A-1372, Molecular Probes-Life Technologies) for 15 minutes. Afterwards cells were centrifuged as before at 4°C, washed and transferred into FACS buffer for flow cytometry measurements on Gallios flow cytometer (Beckman-Coulter, Miami, FL, USA).

### Microscopy

#### Confocal microscopy

For immunofluorescence imaging, cells were seeded onto cover slips in 24 wells plates or μ-Slide 8 well plates (ibidi# 80826). When required, mitochondria were stained with MTR (50 nM). MTR was loaded for the last 30 minutes of treatment and before fixation. Cells were fixed with 4% paraformaldehyde, permeabilised with Triton X, blocked with BSA, incubated with the appropriate primary antibodies. Secondary antibody was goat anti-mouse IgG (H+L) Alexa Fluor 488 (Invitrogen, #A00037). After staining the nucleus with DAPI, coverslips were mounted on slides using mounting solution (DakoCytomation #125046). Images were taken with the microscope LSM700 using Plan-Apochromat 63x/1.40 Oil DIC M27 objective lens with a pinhole adjusted at 68 μm.

For mitophagy live imaging, cells seeded onto μ-Slide 8 well plates (ibidi# 80826), were maintained under CO_2_ at 37°C and visualised under the LSM780 microscope using Plan-Apochromat 63x/1.40 Oil DIC M27 lens and pinhole 90 μm. Cell exposure for 2 hours to CCCP (50 μM) served as positive control for mitophagy.

#### Electron microscopy

For transmission electron microscopy pellets of HCT116 cells were fixed with 2.5% glutaraldehyde in 0.1 M cacodylate buffer, pH 7.4, at 4° overnight. Postfixation was done in 1% OsO_4_ for 90 min, followed by dehydration in ethanol and embedding in low viscosity epoxy resin (Agar scientific, UK). Thin sections for ultrastructural assessment, were cut with an Ultracut S ultramicrotome (Leica Microsystems, Austria). Sections were mounted on copper grids, counterstained with uranyl acetate and lead citrate, and examined at 80 kV in a ZEISS Libra 120 transmission electron microscope. Images were acquired by using a TRS camera (Slow Scan CCD) for the bottom port of the TEM and iTEM software (OlympusSoft Imaging Solutions GmbH, Germany).

### Respiration

Oxygen consumption was measured in whole cells using a Clark-type electrode (Rank Brothers, Cambridge UK). In brief, HCT116^WT^ cells were treated with either vehicle or KP46 (2.5 μM) for 1,2,4,6 h then washed, trypsinized and counted, 4 × 10^6^ cells were then suspended in respiration medium (1 mM sodium pyruvate, 25 mM glucose, 2% BSA in PBS, pH 7.4) and the oxygen consumption was measured at 37°C. Uncoupled respiration was determined by the addition of oligomycin (1 μg/mL) after a stable basal rate was obtained, non-mitochondrial respiration was determined by the addition of antimycin A (4 μM) and rotenone (5 μM). ATP turnover was calculated by the subtraction of uncoupled respiration from basal respiration rate.

The Seahorse XF analyzer (Seahorse Bioscience, Billerica, MA) was used to measure extracellular fluxes in a XF24 or XFp well format. For XF24, HCT116^WT^ cells were seeded at a density of 40 000 cells per well in 100 μL growth media with 4 empty control wells. On the day of measurement, the media was changed to growth media supplemented with either DMSO or KP46 (10 μM) for the indicated length of time. After treatment, the cells were washed with XF DMEM assay medium (25 mM glucose, 1 mM pyruvate, pH 7.4), 675 μL XF DMEM assay media was then added and the cells incubated for 1 h at 37°C in a CO_2_ free incubator before measurement. The experiments were similarly conducted in the XFp instrument, with the exception that cells were seeded at a density in 20 000 per well in 50 μL. XF24 or XFp extracellular flux analyzer measured oxygen consumption, FCCP (0.2 μM) or FCCP (0.24 μM), oligomycin (1 μM), antimycin A (0.5 μM) and rotenone (0.5 μM), respectively, were added at the time as indicated. After measurement, when indicated, protein content of all wells was determined by Bradford Assay (Bio-Rad laboratories, Pty, Hercules, California, USA) and used to normalize OCR readings.

### Assessment of the labile iron pool (fluorescent calcein-assay)

The labile iron pool (LIP) was assayed by the fluorescence calcein-assay developed by Breuer et al. [52] with modifications for adherent cells described by Sturm et al. [53]. For the fluorescent calcein-assay, HCT116^WT^ cells, cultivated in McCoy's 5A culture medium, were incubated with KP46 (2.5 μM) for the indicated times and then washed with culture medium without FBS containing diethylenetriaminepentaacetic acid (DTPA) (50 μM) and twice in medium without FBS to remove surface bound iron. Following loading the cells with calcein-AM (0.25 μM) in 20 mM Hepes buffered serum-free growth medium for 15 minutes at 37°C and washings, they were incubated with medium with anti-calcein antibody and 20 mM Hepes. Anti-calcein antibody was prepared according to a protocol of William Breuer (Hebrew University of Jerusalem, Israel) as described by [54]. The anti-calcein antibody was used to quench fluorescence of extracellular calcein. Measurement was performed at Ex485nm/Em535nm (measurement A) in a fluorescence plate reader (Anthos Zenyth 3100, HVD Vienna). Three minutes after addition of salicylaldehyde isonicotinoyl hydrazine (SIH) (100 μM), a strong iron chelator, the plate was measured again (measurement B). The difference between measurement B and measurement A represents the LIP. Shown is the total fluorescence intensity. Exact concentrations could not be obtained reliably because the cell-free calibration and the assessment in the cellular system were apparently not exactly equal.

### Heme measurement

Heme content was measured fluorometrically using the fluorescence of protoporphyrin IX as outlined in [55] with minor modification. In brief, cells were treated as indicated with DMSO or KP46, washed and then harvested in 1% Triton X in PBS. Cellular debris was removed by centrifugation at 2000 *g* for 10 minutes; protein concentration of the supernatant was determined by Bradford assay (Bio-RAD, Pty, Hercules, California, USA). Lysates (100 μg protein) were boiled in 2 M oxalic acid for 30 minutes. The samples were diluted in 2X volume water and fluorescence was measured using a plate reader at Ex405nm/Em600nm (TristarLB941, Berthold Technologies). For each experiment a standard curve was generated using hemin at concentrations ranging between 0-600 pmol.

### Calcium retention capacity

The CRC assay was performed according to [56]. Briefly, 7×10^6^ cells were trypsinised, resuspended and centrifuged at 600 *g* for 7 min. at 4°C. The cell pellet was washed in isotonic buffer IB (10 mM Tris/Mops, 1 mM Pi-Tris, 130 mM KCl and 0.1 mM EGTA/Tris, pH 7.4) and permeabilized using digitonin (150 μM) for 15 min, at 4°C in presence of 1 mM EGTA. Cells were washed once in IB containing 0.1 mM EGTA and resuspended in IB containing 10 μM EGTA in the presence of Calcium-Green-5N (1 μM), cytochrome C (10 μM) and succinate (5 mM) for fluorometric measurements. Cells were exposed to Ca^2+^ spikes (10 μM) and the Calcium-Green-5N fluorescence was measured at 25°C with the fluorescence spectrometer (Ex505 nm/Em535 nm) using LS50B (Perkin Elmer, Waltham, MA).

## SUPPLEMENTARY MATERIALS FIGURES



## Abbreviations

KP46: tris(8-quinolinolato)gallium(III)
BNIP3L: BCL2/adenovirus E1B 19kDa interacting protein 3-like
ATG: autophagy-related
VDAC1: voltage-dependent anion channel 1
Bax: Bcl-2 associated X protein
BID: Bax-like BH3 protein
PUMA: p53 upregulated modulator of apoptosis
PTP: permeability transition pore
MPT: mitochondrial permeability transition
TEM: transmission electron mitcroscopy
MTR: MitoTracker Red
LC-3: Microtubule-associated protein 1A/1B-light chain 3
*mt*RFP: mitochondrial targeted red fluorescent protein
ND6: NADH dehydrogenase 6
COX4: Cytochrome c oxidase subunit 4
HSP60: heat-shock protein 60
OCR: oxygen consumption rate
FCCP: carbonyl cyanide-4-(trifluomethoxy) phenylhydrazone
LIP: labile iron pools
CCCP: carbonyl cyanide m-chlorophenylhydrazone
PGC1a: peroxisome proliferator-activated receptor gamma, coactivator 1 alpha
TMRM: tetramethylrhodamine methyl ester perchlorate
MitoSOX: Red mitochondrial superoxide indicator
ROS: reactive oxygen species
MPT: mitochondrial permeability transition
CRC: calcium retention capacity
CsA: Cyclosporin A
Cyp-D: Cyclophilin-D
ER: endoplasmatic reticulum
7AAD: 7-Aminoactinomycin-D
NAO: Nonyl acridine orange (Acridine Orange 10-Nonyl Bromide)
∆ψ_m_: mitochondrial membrane potential
ns: non significant
SR: sarcoplasmic reticulum
SERCA: Sarcoplasmic/endoplasmic reticulum calcium ATPase
