# Supplementary material for: Novel p53-dependent anticancer strategy by targeting iron signaling and BNIP3L-induced mitophagy
Source: Oncotarget. 2015 Oct 26;7(2):1242–61. doi: 10.18632/oncotarget.6233 (PMC4811457; doi:10.18632/oncotarget.6233)
Supplement: Supplementary file 1 [file oncotarget-07-1242-s001.pdf]

Novel p53-dependent anticancer strategy by targeting iron signaling and BNIP3L-induced mitophagy

Supplementary Materials

Detection of KP46 autofluorescence

Cells were seeded onto glass bottom plates (34.8 mm), transfected with mtRFP and exposed to KP46 (100 µM) for 30 minutes. Fluorescence microscope BX40 with F-View CCD Camera, Cell^F fluorescence imaging software and 60x magnification oil immersion objective lens (all from Olympus, Vienna, Austria) were used. The fluorescence filter cubes U-MWU2 (UV excitation) and U-MWG2 (green light excitation) were used to detect KP46 and mtRFP, respectively. Imaging settings were adjusted before every photo to exclude overlapping.

Flow cytometry

To determine the mitochondrial membrane potential, cells exposed to vehicle or KP46 (10 µM) for the indicated periods of time were stained for 30 minutes with TMRM (100 nM). Exposure to Valinomycin (200 nM) for 30 minutes at 37°C served to generate depolarised controls. Cells were washed with PBS, trypsinised and resuspended in fresh growth medium. After two washing steps, the TMRM fluorescence intensity was immediately visualized by flow cytometer on FL2.

To measure mitochondrial superoxide production, MitoSOX Red was used after titration of the appropriate concentration to exclusively stain mitochondria. Cells exposed to vehicle or KP46 (10 µM) for 2, 4, 8, 24, 48 or 72 hours were stained for 30 minutes with MitoSOX (4 µM) Red. Exposure of control cells to Antimycin A (50 µM) for 30 minutes at 37°C served as positive control. After two washing steps the fluorescence intensity of MitoSOX Red was visualized by the flow cytometer in FL2.

Viability assay

Cell viability of HEK293T and HCT116^{WT} cells was assessed using the CellTiter-Blue Cell Viability Assay (Promega Madison, WI, USA # G8081). Cells were seeded onto 96 wells plate (5000 cells/well) and exposed the next day to DMSO or KP46 as indicated for 48 hours. For the last 2 hours cells were loaded with CellTiter-Blue solution according to the manufactures protocol and the fluorescence recorded at Ex560/Em590nm using a micro plate reader (TriStar LB941, Berthold Technologies).

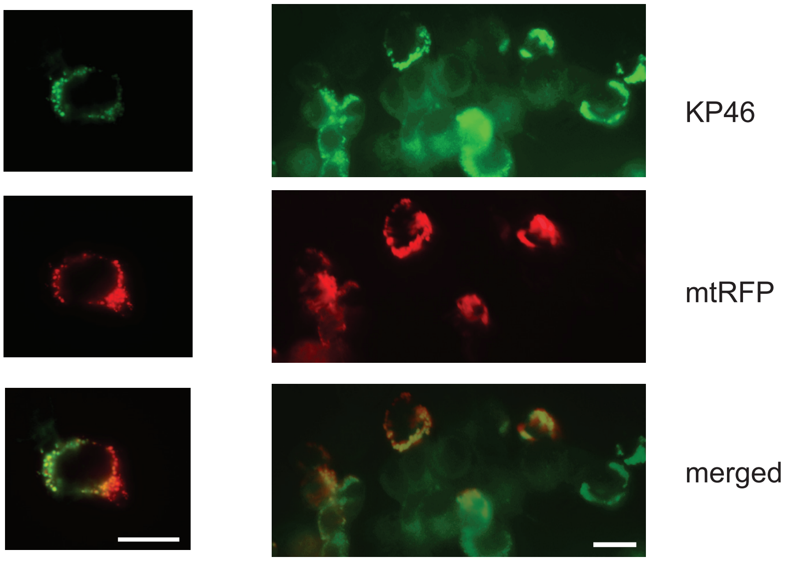

Supplementary Figure S1: KP46 is specifically delivered to mitochondria. HCT116^{WT} cells transiently expressing mtRFP were exposed to KP46 at high doses for enhanced fluorescence signal and the fluorescence immediately monitored under the microscope. Live imaging reveals the colocalization of the fluorescence of KP46 and mtRFP in mtRFP transfected cells, while untransfected cells only displayed the autofluorescence of KP46. Note that the high dose of KP46 also caused the immediate fragmentation of mitochondrial tubules into globular units. Scale bars: 20 µm.

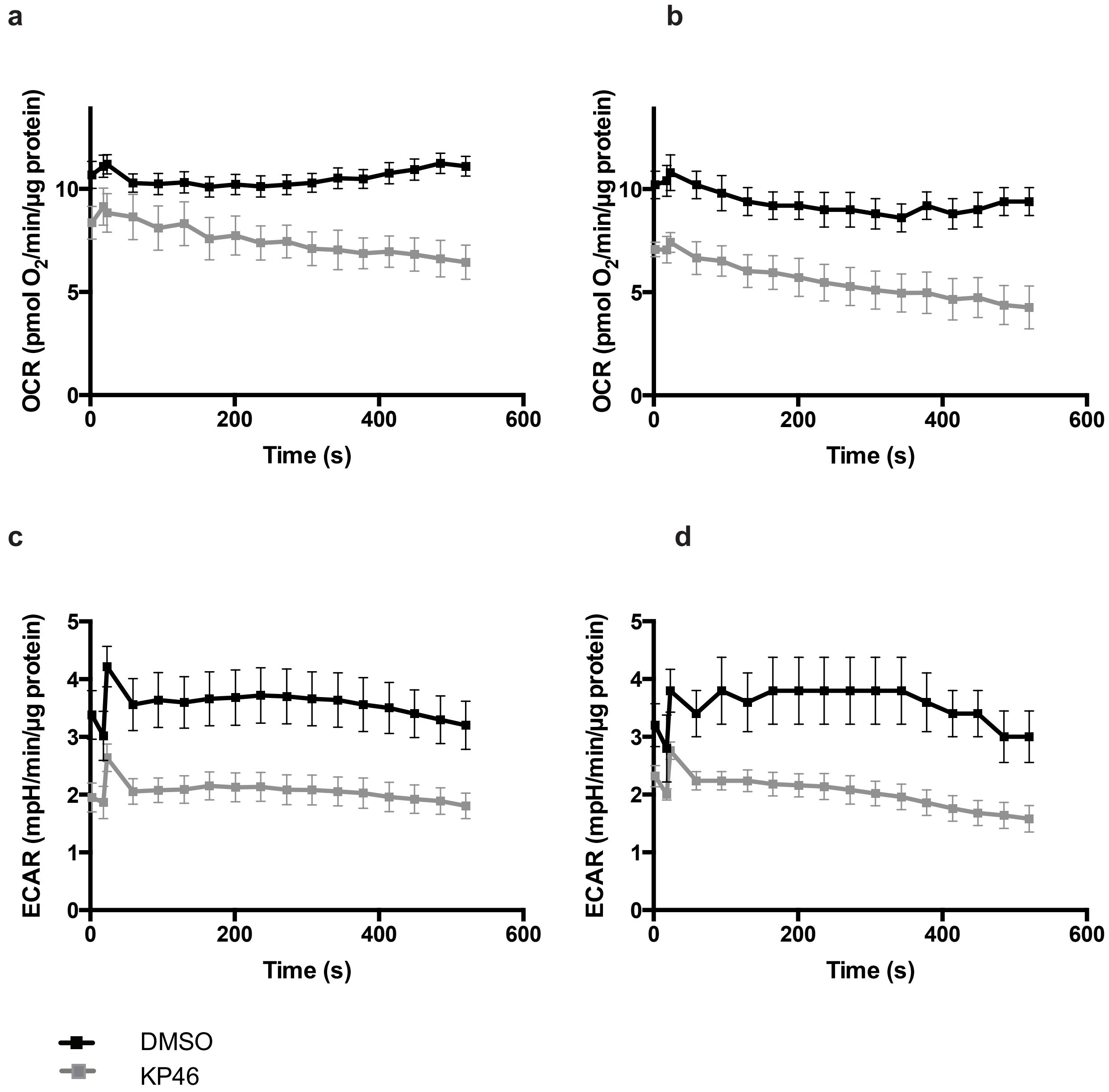

Supplementary Figure S2: KP46 lowers OCR and ECAR. a–b. Oxygen consumption rate (OCR) for HCT116^{WT} cells treated for 4 h (a) or 6 h (b) with KP46 (2.5 µM). **c–d** extracellular acidification rate (ECAR) for HCT116^{WT} cells treated for 4 h (c) or 6 h (d) with KP46 (2.5 µM). Black lines indicate DMSO control, grey lines KP46 treated. Data are means, error bars represent ±SEM (*n* = 3).

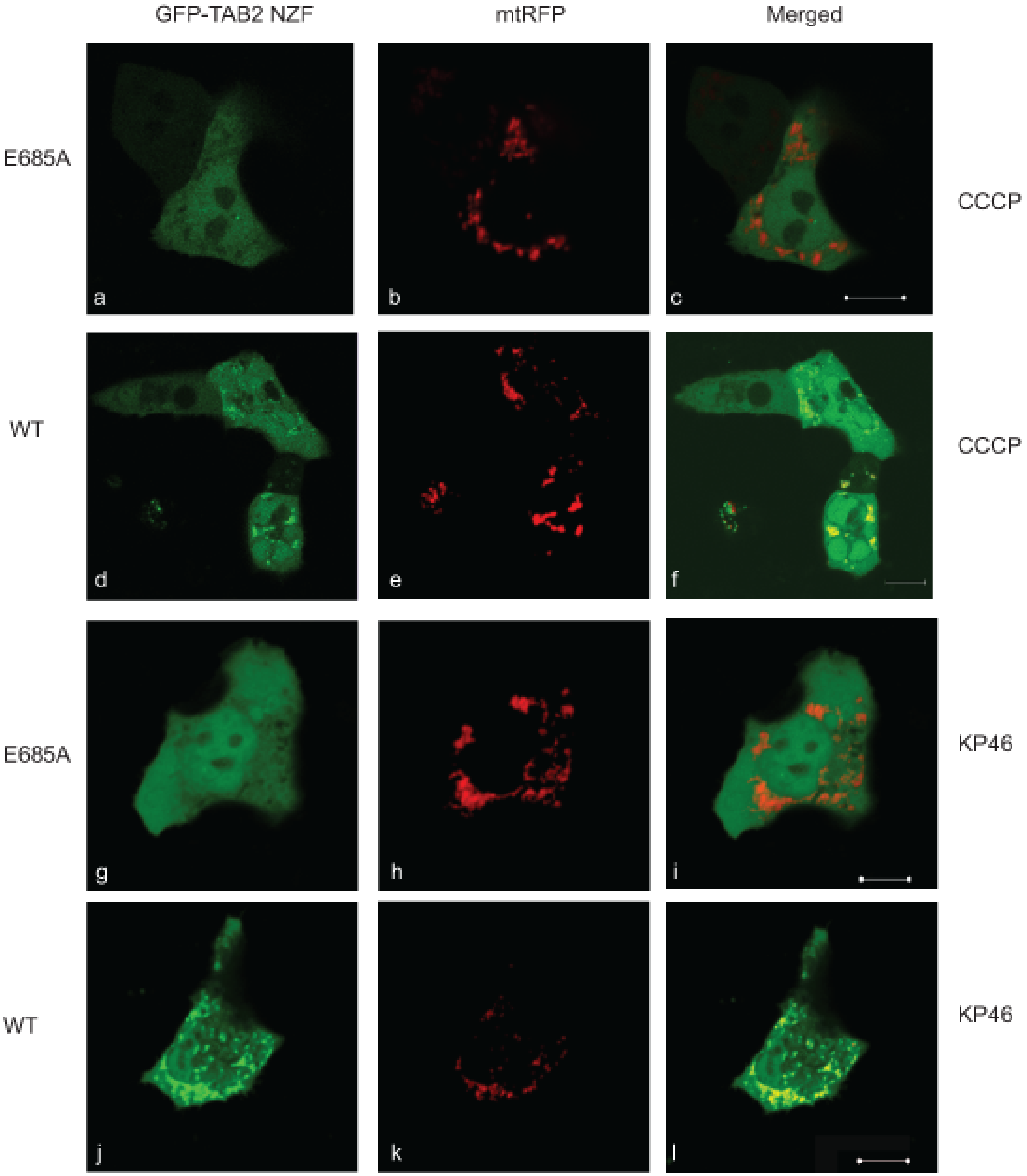

Supplementary Figure S3: KP46-treated mitochondria are K63 ubiquitinated. Confocal microscopy of HCT116 cells expressing mtRFP (red) and GFP-TAB2 NZF (green) (indicated in the figure legend left side as WT) or GFP-TAB2 ^{E685A} NZF (indicated in the figure legend left side as E685A) treated with CCCP for 3 hours or KP46 for 4 hours. Bars: 10 µm.

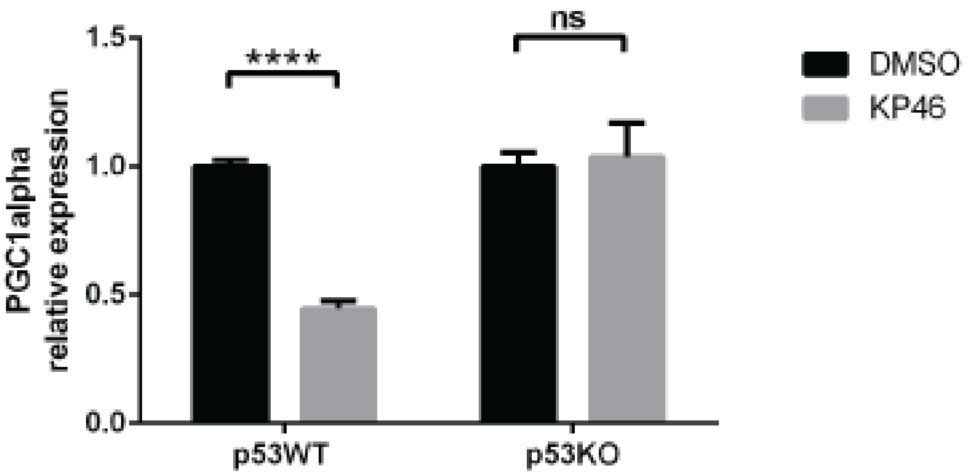

Supplementary Figure S4: KP46 represses PGC1α in the presence of p53. PGC1α transcripts were analysed in $HCT116^{WT}$ and $HCT116p53^{KO}$ cells exposed to vehicle or 10 μM KP46 (grey or black bars, respectively) for 4 hours and shown as mean normalized expression (*n* = 3 independent measurements, carried out as triplicates). ****p < 0.001, two-way ANOVA followed by Bonferroni's multiple comparisons test.

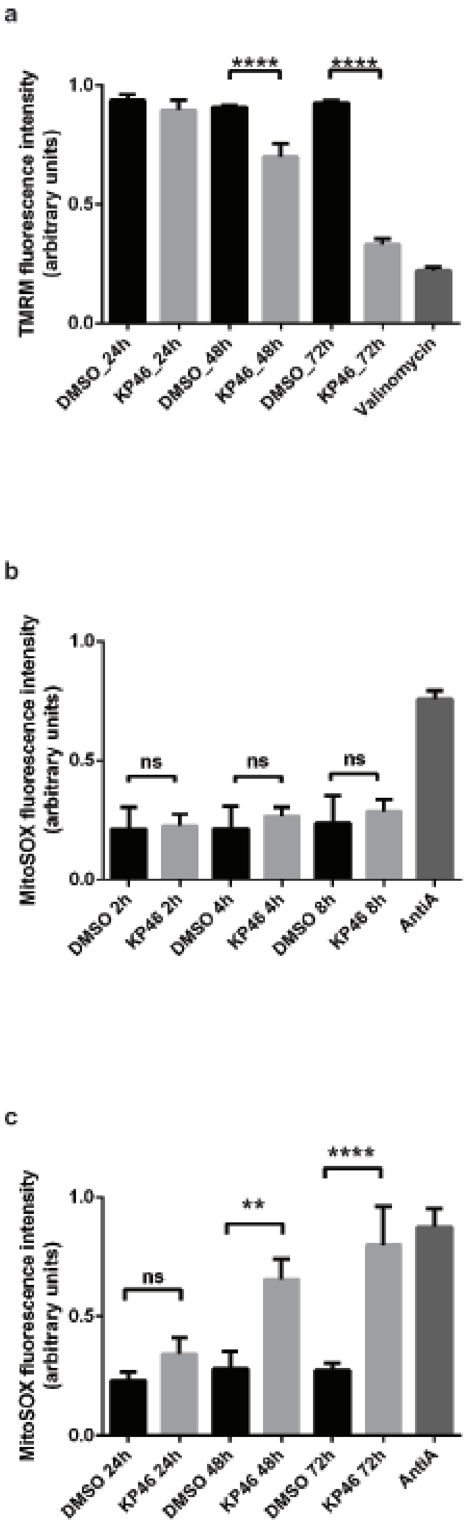

Supplementary Figure S5: KP46 does not affect mitochondrial membrane potential (Δψ) or ROS at early stage. a–c. $\Delta\psi_m$ and superoxide formation a.Flow cytometric analyses of the changes of the TMRM fluorescence of $HCT116^{WT}$ cells treated with DMSO or 10 µM KP46 for 24, 48 and 72 hours or 200 nM Valinomycin for 30 minutes. Shown are the relative fluorescence intensities ±SD (n = 3). ****p < 0.0001, one-way ANOVA followed by Tukey‘s multiple comparison test. **b–c.** Flow cytometry recording of the changes of the fluorescence of MitoSOX after 2, 4 and 8 hours exposure to vehicle or 10 µM KP46 (b). MitoSOX fluorescence changes were also recorded after 24, 48 or 72 hours (c). Antimycin A (AntiA) served as positive control in (b–c). Shown are the relative fluorescence intensities ±SD (n = 3). ****p < 0.0001, **p < 0.01, one-way ANOVA followed by Tukey‘s multiple comparison test.

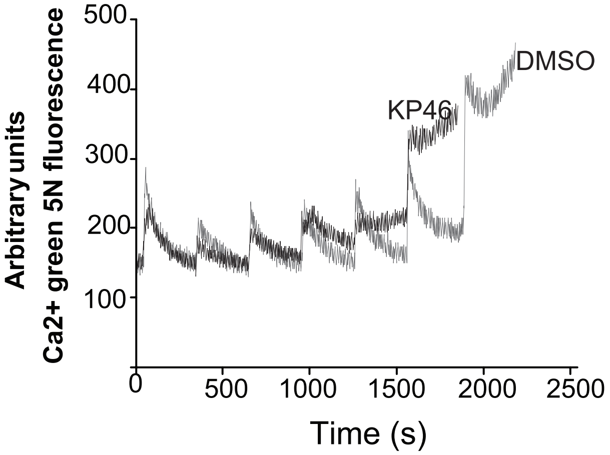

Supplementary Figure S6: p53 controls the sensitivity of the PTP to Ca^{2+} pulses. CRC assay as described in Figure 8 was performed on HCT116p53^{KO} cells treated with 2.5 μM KP46 or DMSO for 4 h.

a

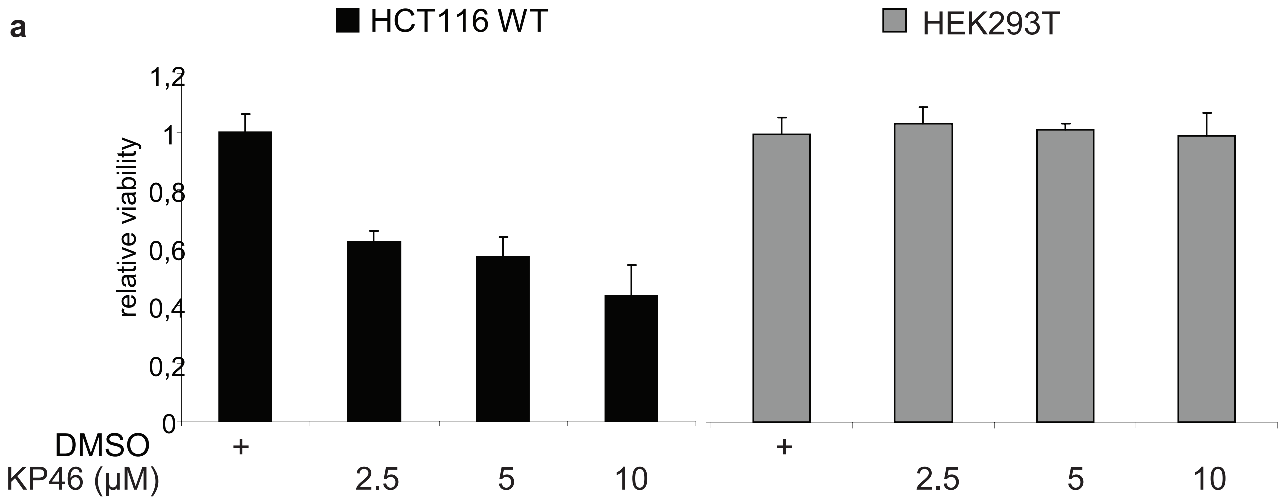

b

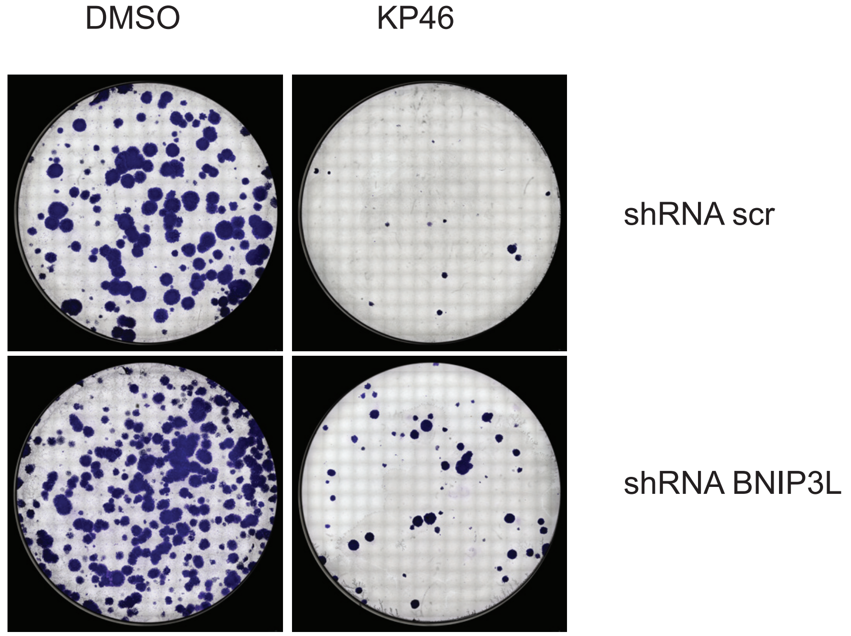

Supplementary Figure S7: KP46 affects HCT116^{WT} cells but not control cells. a. Cell viability was measured after exposure to DMSO or 2.5 µM, 5 µM or 10 µM KP46 for 48 hours. Shown is the relative viability of HEK293T and HCT116^{WT} cells. **b.** Representative images of clonogenic assay. Colony formation of HCT116^{WT}shRNAscr and HCT116shRNABNIP3L^{KD} cells treated with vehicle or 2.5 µM KP46 for 48 hours, seeded at a density of 1000 cells/well (6-wells plate) and cultured for 14 days.
